# Molecular determinants of response to neoadjuvant pembrolizumab plus chemotherapy in patients with high-risk, early-stage, triple-negative breast cancer: exploratory analysis of the open-label, multicohort phase 1b KEYNOTE-173 study

**DOI:** 10.1186/s13058-024-01946-y

**Published:** 2025-03-11

**Authors:** Rebecca Dent, Javier Cortés, Yeon Hee Park, Eva Muñoz-Couselo, Sung-Bae Kim, Joohyuk Sohn, Seock-Ah Im, Esther Holgado, Theodoros Foukakis, Sherko Kümmel, Jennifer Yearley, Anran Wang, Michael Nebozhyn, Lingkang Huang, Razvan Cristescu, Petar Jelinic, Vassiliki Karantza, Peter Schmid

**Affiliations:** 1 Division of Medical Oncology, National Cancer Centre Singapore, 30 Hospital Blvd, Singapore, 168583 Singapore; 2 Vall d´Hebron Institute of Oncology (VHIO), Barcelona, Spain; 3 International Breast Cancer Center, Quironsalud Group, Barcelona, Spain; 4 Department of Medicine, Faculty of Biomedical and Health Sciences, European University of Madrid, Madrid, Spain; 5 Department of Medicine, Division of Hematology-Oncology, Samsung Medical Center, Sungkyunkwan University School of Medicine, Seoul, Republic of Korea; 6 Department of Medical Oncology, Vall d’Hebron Hospital, Barcelona, Spain; 7 Department of Oncology, Asan Medical Center, University of Ulsan College of Medicine, Seoul, Republic of Korea; 8 Department of Internal Medicine, Division of Medical Oncology, Yonsei Cancer Center, Yonsei University College of Medicine, Seoul, Republic of Korea; 9 Department of Internal Medicine, Cancer Research Institute, Seoul National University Hospital, Seoul National University College of Medicine, Seoul, Republic of Korea; 10 Medical Oncology Service, Ramón y Cajal University Hospital, Madrid, Spain; 11 Department of Oncology-Pathology, Karolinska Comprehensive Cancer Center, Karolinska Institute and Breast Cancer Centre, Cancer Theme, Karolinska University Hospital, Solna, Sweden; 12 Interdisciplinary Breast Unit, Essen-Mitte Clinics, Essen, and Charité–Universitätsmedizin Berlin, Berlin, Germany; 13 Merck & Co., Inc., Rahway, NJ USA; 14 Centre for Experimental Cancer Medicine, Barts Cancer Institute, London, UK

**Keywords:** Immunohistochemistry, Triple-negative breast cancer, Tumor microenvironment

## Abstract

**Background:**

The multicohort, open-label, phase 1b KEYNOTE-173 study was conducted to investigate pembrolizumab plus chemotherapy as neoadjuvant therapy for triple-negative breast cancer (TNBC). This exploratory analysis evaluated features of the tumor microenvironment that might be predictive of response.

**Methods:**

Cell fractions from 20 paired samples collected at baseline and after one cycle of neoadjuvant pembrolizumab prior to chemotherapy initiation were analyzed by spatial localization (tumor compartment, stromal compartment, or sum of tumor and stromal compartments [total tumor]) using three six-plex immunohistochemistry panels with T-cell, myeloid cell, and natural killer cell components. Area under the receiver operating characteristic curve (AUROC) was used to assess associations between immune subsets and gene expression signatures (T-cell–inflamed gene expression profile [Tcell_inf_GEP] and 10 non-Tcell_inf_GEP signatures using RNA sequencing) and pathologic complete response (pCR).

**Results:**

At baseline, six immune subsets quantitated within the tumor compartment showed AUROC with 95% CIs not crossing 0.5, including CD11c^+^ cells (macrophage and dendritic cell [DC]: AUROC, 0.85; 95% confidence interval [CI] 0.63–1.00), CD11c^+^/MHCII^+^/CD163^−^/CD68^−^ cells (DC: 0.76; 95% CI, 0.53–0.99), CD11c^+^/MHCII^−^/CD163^−^/CD68^−^ cells (nonactivated/immature DC: 0.80; 95% CI 0.54–1.00), and CD11c^+^/CD163^+^ cells (M2 macrophage: 0.77; 95% CI 0.55–0.99). Other associations with pCR included baseline CD11c^+^/MHCII^−^/CD163^−^/CD68^−^ (nonactivated/immature DC) within the total tumor (AUROC, 0.76; 95% CI 0.51–1.00) and the baseline CD11c/CD3 ratio within the tumor compartment (0.75; 95% CI 0.52–0.98). Changes in immune subsets following one cycle of pembrolizumab were not strongly associated with pCR. Although T-cell associations were relatively weak, specific CD8 subsets trended toward association. The AUROC for discriminating pCR based on Tcell_inf_GEP was 0.55 (95% CI 0.25–0.85); when detrended by Tcell_inf_GEP, AUROC varied for the non-Tcell_inf_GEP signatures. Tcell_inf_GEP expression trended higher in responders than in nonresponders when evaluating pCR.

**Conclusions:**

Myeloid cell populations within the tumor compartment at baseline and Tcell_inf_GEP show a promising trend toward an association with pCR in a small subgroup of patients with early-stage TNBC treated with neoadjuvant pembrolizumab plus chemotherapy.

**Trial registration:**

ClinicalTrials.gov, NCT02622074; registration date, December 2, 2015.

**Supplementary Information:**

The online version contains supplementary material available at 10.1186/s13058-024-01946-y.

## Background

Triple-negative breast cancer (TNBC) is a heterogeneous disease with distinct pathological, genetic, and clinical features among molecular subtypes that result in different prognoses and varying sensitivity to neoadjuvant treatment [1–3]. Poor long-term outcomes have been observed in patients with TNBC compared with non-TNBC, despite initial sensitivity to chemotherapy [4, 5]. Among patients with advanced TNBC, treatment with immune checkpoint inhibitors early in the disease course has been associated with better response rates than treatment later in the disease course, likely due to the development of immune escape mechanisms driven by features of the tumor microenvironment (TME) [6–11]. The features of the TME that may influence clinical response to immune checkpoint inhibitors are not well understood.

Recent evidence supports the addition of programmed cell death protein 1 (PD-1) or programmed death ligand 1 (PD-L1) inhibitors to neoadjuvant chemotherapy for high-risk, early-stage TNBC [12–17]. In the phase 1b KEYNOTE-173 study, neoadjuvant pembrolizumab plus chemotherapy showed manageable toxicity and promising antitumor activity in high-risk early-stage TNBC (pathologic complete response [pCR], 60%; range, 49–71%). Higher baseline PD-L1 combined positive score (CPS) and pre- and on-treatment stromal tumor-infiltrating lymphocytes were significantly associated with higher pCR rates (*P* = 0.0127, 0.0059, and 0.0085, respectively) [13]. In the phase 3 KEYNOTE-522 study, the benefit of neoadjuvant pembrolizumab plus chemotherapy with respect to differences in pCR was observed in patients with early-stage TNBC with high (PD-L1 CPS ≥ 1; 14.2%; 95% CI 5.3–23.2) and low PD-L1 expression (PD-L1 CPS < 1; 18.3%; 95% CI − 3.3 to 36.8); a statistically significant improvement in pCR (estimated treatment difference, 13.6%; 95% CI 5.4–21.8; *P* < 0.001) was also observed in patients with PD-L1 CPS ≥ 1 tumors [12]. After 63.1 months of follow-up, a clinically meaningful improvement in event-free survival was observed with neoadjuvant pembrolizumab plus chemotherapy followed by adjuvant pembrolizumab compared with neoadjuvant chemotherapy alone (hazard ratio [HR], 0.63; 95% CI 0.49–0.81) [18].

Molecular characterization of early-stage TNBC may help identify patients who have a pCR after neoadjuvant therapy with pembrolizumab plus chemotherapy. Although higher expression of PD-L1, T-cell–inflamed gene expression profile (Tcell_inf_GEP), tumor mutational burden, CD8^+^ T cells, and stromal tumor-infiltrating lymphocytes have been associated with improved response to pembrolizumab monotherapy in the metastatic setting [19, 20], the association between features of the TME and clinical efficacy with neoadjuvant pembrolizumab are not well understood. MHCII expression has been associated with response to PD-(L)1 inhibition across multiple malignancies, such as melanoma, classic Hodgkin lymphoma, and bladder cancer [21–24]. In TNBC, MHCII expression was shown to be positively correlated with T cell expansion following treatment with a PD-1 inhibitor [25]. In a multivariate model of TME features using biopsy samples from patients with TNBC enrolled in the NeoTRIP trial, MHCII-positive cancer cells were predictors of response to atezolizumab plus neoadjuvant chemotherapy [26]. In a phase 1/2 study, MHCII was predictive of response to pembrolizumab plus neoadjuvant chemotherapy in HER2-negative breast cancer, but the predictive role of MHCII in TNBC remained unclear [27].

Multiplex immunohistochemistry (mIHC) is an investigative tool that provides objective quantitative data by analyzing multiple biomarkers to describe the TME based on immune subsets and tumor compartments. To improve the understanding of the TME and drivers of response to neoadjuvant pembrolizumab plus chemotherapy in high-risk early-stage TNBC, we explored clinical response to neoadjuvant pembrolizumab plus chemotherapy and the effects of a single cycle of pembrolizumab on immune subsets based on tumor compartments using the novel mIHC methodology and gene expression signatures using RNA sequencing in patients enrolled in KEYNOTE-173.

## Methods

### Patients and study design

KEYNOTE-173 (NCT02622074) was an international, open-label, multicohort, phase 1b study designed to evaluate regimens of pembrolizumab plus chemotherapy as neoadjuvant treatment in adult women with newly diagnosed, early-stage, high-risk TNBC, defined as estrogen receptor/progesterone receptor–negative and HER2-negative by in situ hybridization or immunohistochemistry. Eligibility criteria and the study design have been previously described [13]. Briefly, patients had previously untreated, nonmetastatic (M0) disease (T1c, N1–N2; T2–T4c, N0–N2) according to American Joint Committee on Cancer staging, 7th Edition [28] and an Eastern Cooperative Oncology Group performance status of 0 or 1.

The study enrolled six cohorts for treatment with different pembrolizumab plus chemotherapy regimens. All patients received pembrolizumab 200 mg every 3 weeks (Q3W) intravenously before surgery for 9 cycles or until unacceptable toxicity or withdrawal of consent. In cycles 2 through 5, patients also received weekly nab-paclitaxel 125 mg/m^2^ (cohort A), weekly nab-paclitaxel 100 mg/m^2^ plus carboplatin area under the curve (AUC) 6 Q3W (cohort B), weekly nab-paclitaxel 125 mg/m^2^ plus carboplatin AUC5 Q3W (cohort C), weekly nab-paclitaxel 125 mg/m^2^ plus weekly carboplatin AUC2 (cohort D), weekly paclitaxel 80 mg/m^2^ plus carboplatin AUC5 Q3W (cohort E), or weekly paclitaxel 80 mg/m^2^ plus weekly carboplatin AUC2 (cohort F). In cycles 6 through 9, all patients received doxorubicin 60 mg/m^2^ plus cyclophosphamide 600 mg/m^2^ Q3W. Definitive surgery was performed 3 to 6 weeks after completion of neoadjuvant treatment.

The study protocol and all amendments were approved by the institutional review board or ethics committee at each participating institution. The study was conducted in accordance with the protocol, its amendments, the ethical principles originating from the Declaration of Helsinki, and Good Clinical Practice guidelines. Written informed consent was provided by all patients before enrollment.

### Assessments

Tumor tissue samples were obtained at baseline and after 1 cycle of pembrolizumab prior to initiation of chemotherapy. Three six-plex immunohistochemistry panels were evaluated: natural killer cell panel (CD16/CD56/CD11b/CD20/CD3/CD45), activated T-cell panel (CD3/CD8/FoxP3/Ki67/granzyme B/PD-1), and myeloid cell panel (CD68/CD163/MHCII/arginase/CD33/CD11c). These panels were used to quantify TME-associated cell fractions (B cells, natural killer cells, total T cells, Treg, activated and inactive CD8 T cells, dendritic cells [DCs], granulocytes, and total and M2 macrophages) by spatial localization (tumor compartment, stromal compartment, and sum of tumor plus stromal compartments [total tumor]) on whole-slide images with Halo software (Indica Labs, Albuquerque, NM, USA). For mIHC, formalin-fixed, paraffin-embedded tissue blocks were used to cut 5-μm sections that were baked at 60 °C for 1 h then deparaffinized and rehydrated with xylene and graded ethanols. Thereafter, slides were subjected to heat-induced epitope retrieval in 1× target retrieval solution (Agilent, Santa Clara, CA, USA). Slides were then incubated in 3% hydrogen peroxide solution to block endogenous peroxidase followed by PKI buffer to block protein (Akoya Biosciences, Marlborough, MA, USA). A Bond RX stainer (Leica Biosystems, Buffalo Grove, IL, USA) was used for six-plex staining with tyramide signal amplification–based Opal multiplexing reagents (Akoya Biosciences). Each primary antibody (Supplementary Table 1) was incubated for 60 min, followed by Opal polymer horseradish peroxidase and tyramide signal amplification–conjugated Opal fluorophore application (Akoya Biosciences). Antibodies were stripped using epitope retrieval 1 buffer (Leica Biosystems) after each staining cycle. Nuclei were detected using Spectral DAPI (Akoya Biosciences), and slides were cover-slipped for scanning. Stained slides were scanned using the Vectra 3 Imaging System (Akoya Biosciences) at 20× magnification. Image tiles were deconvoluted using inForm software (Akoya Biosciences) and stitched into whole-slide images using Halo software (Indica Labs, Albuquerque, NM, USA), followed by quantitative analysis. Manual annotations were used to define tumor-containing regions on stitched mIHC images in Halo software (version 3.0.311.337) with the reference of serial hematoxylin and eosin scans. Tumor-containing regions were segmented into tumor and stroma compartments using the Random Forest classifier in Halo. Density, defined as number of positive cells per mm^2^, was calculated for each analyte in the tumor compartment, stromal compartment, and total tumor.

RNA sequencing was performed using the HiSeq 3000/HiSeq 4000 platform (Illumina Inc, San Diego, CA, USA). The RNA-sequencing raw reads were processed using OmicSoft sequence aligner (OmicSoft ArraySuite, Qiagen, Germantown, MD, USA) and were aligned to the reference genome Human.B37.3 followed by quantification with Ensembl.R75 as the genome model. The Tcell_inf_GEP status was derived from the weighted sum of the normalized expression value of the 18-gene signature present on the NanoString Pan Cancer Immune Panel run at Almac Diagnostic Service [29]. Scores for the 10 non-Tcell_inf_GEP signatures (angiogenesis, glycolysis, granulocytic myeloid-derived suppressor cell [gMDSC], hypoxia, monocytic myeloid-derived suppressor cell [mMDSC], MYC, proliferation, RAS, stroma/epithelial mesenchymal transition [EMT]/transforming growth factor β [TGFβ], WNT]) were also evaluated [30].

### Objectives and outcomes

In this exploratory analysis, the key objective was to characterize the TME changes and gene expression after one cycle of pembrolizumab and correlation with clinical outcomes. The primary clinical end point of this analysis was pCR rate, defined as no invasive residual disease in breast or nodes, non-invasive breast residuals allowed (pCR_ypT0/Tis ypN0_) or no invasive or non-invasive residual disease in breast or nodes (pCR_ypT0 ypN0_), at the time of definitive surgery (approximately 3 to 6 weeks after completion of neoadjuvant treatment) by local pathology review of biopsied tissue samples. Objective response rate (ORR) was assessed at baseline, at cycle 5, and at the end of treatment, and was evaluated using RECIST v1.1 by investigator review as a secondary end point.

### Statistical analyses

The analysis population comprised all patients enrolled in the KEYNOTE-173 study who received ≥1 dose of pembrolizumab and had matched tissue samples available for response evaluation, mIHC, and RNA sequencing. All cohorts were pooled for this analysis. Responders included patients with pCR by pathologic response assessment or patients who achieved a CR or PR by radiographic response assessment. Nonresponders included patients with no pCR or patients who did not achieve a CR or PR. Response was evaluated based on immune subsets, spatial localization, T-cell population, and Tcell_inf_GEP and non-Tcell_inf_GEP consensus signatures. The area under the receiver operator characteristic curve (AUROC) was used to assess associations between response and immune subsets within the tumor compartment, stromal compartment, and the total tumor at baseline. All analyses were descriptive. Top-ranked findings are reported and were defined as an AUROC (95% CI) that did not cross 0.5, which was considered indicative of correlation with response. The baseline ten non-Tcell_inf_GEP signatures were detrended by Tcell_inf_GEP. The baseline-detrended change from baseline after one cycle of pembrolizumab was evaluated for the six-plex panels by each tumor compartment, Tcell_inf_GEP, and the ten non-Tcell_inf_GEP signatures.

## Results

The median follow-up duration was 19.6 months (range, 4.0–27.4) for 60 patients enrolled across all cohorts. Baseline characteristics of the KEYNOTE-173 study have been previously reported [13]. Briefly, the median age was 48.5 years (range, 26–71); most patients had an Eastern Cooperative Oncology Group performance status of 0 (88%), a primary single lesion (80%), and invasive ductal histology (83%). Of the 60 patients enrolled in the KEYNOTE-173 study, 20 patients who were treated with ≥ 1 dose of pembrolizumab had matched tissue samples available for response evaluation, mIHC, and RNA sequencing. Of the patients included in this analysis, a pCR was observed in 13 patients (65%) and the ORR was 80% (n = 16).

**Fig. 1:**
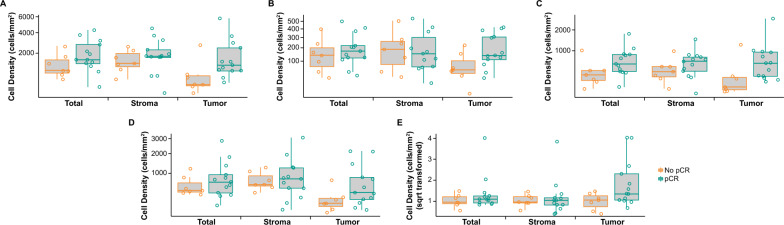
pCR based on immune subsets and spatial localization at baseline. **A** CD11c^+^ (macrophages and DCs); **B** CD11c^+^/MHCII^+^/CD163^−^/CD68^−^ (DCs); **C** CD11c^+^/MHCII^−^/CD163^−^/CD68^−^ (nonactivated/immature DCs); **D** CD11c^+^/CD163^+^ (M2 macrophages); and **E** CD11c/CD3 baseline ratio. Abbreviations: DC: dendritic cell; pCR: pathologic complete response; sqrt: square root

**Fig. 2:**
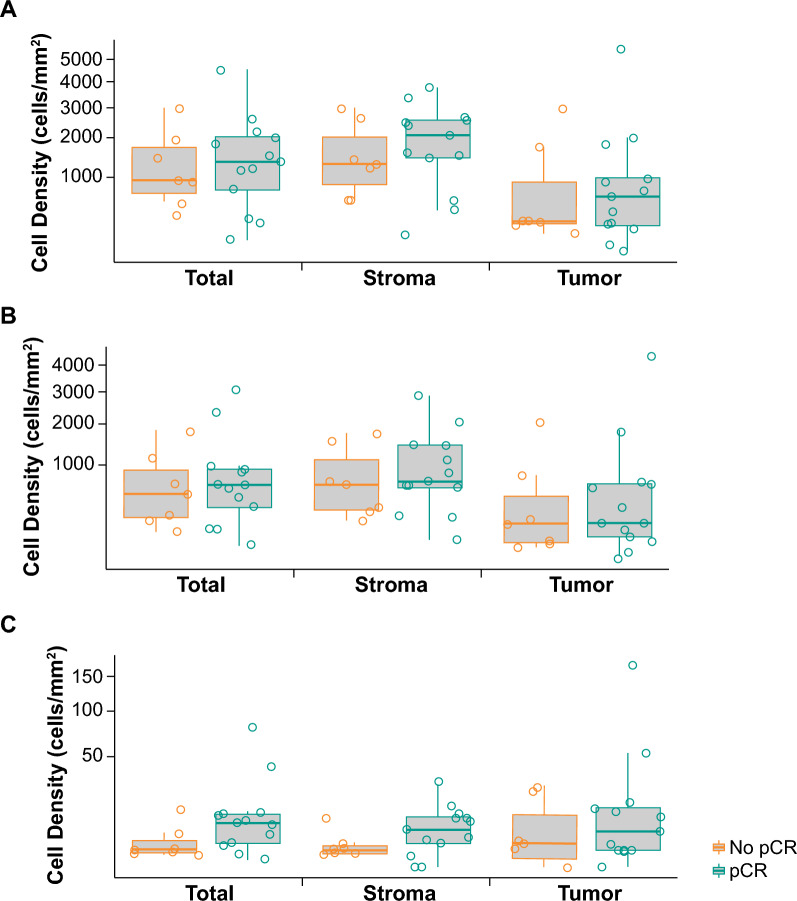
pCR based on T-cell population and spatial localization at baseline. **A** CD3^+^; **B** CD8^+^; and **C** CD8^+^/Granzyme B^+^/Ki67^+^. Abbreviation: pCR: pathologic complete response

**Fig. 3:**
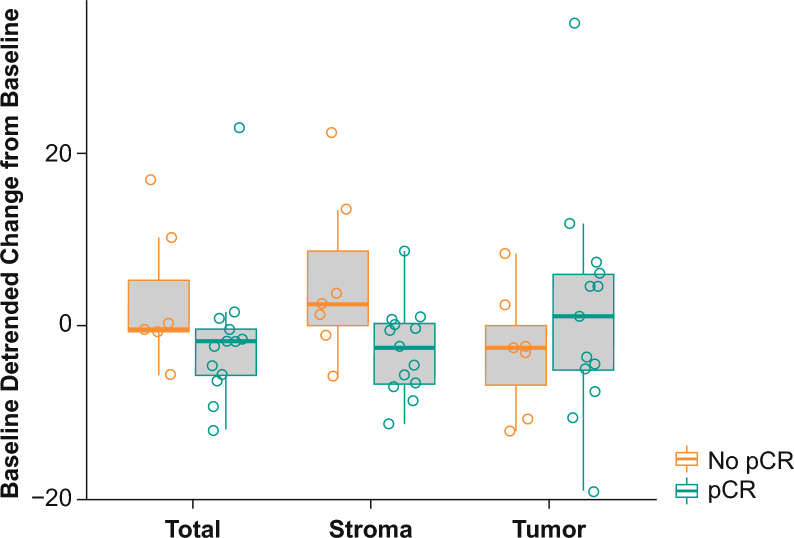
CD163^+^/MHCII^+^ change from baseline detrended by baseline based on pCR and by spatial localization. Abbreviation: pCR: pathologic complete response

Immune cell populations were quantitated as a function of the tissue compartment within which they were localized, yielding 75 immune cell/tissue compartment pairs. Of these 75 immune cell/tissue compartment pairs, the AUROC (95% CI) for discriminating pCR by baseline biomarker status did not cross 0.5 in six immune cell/tissue compartment pairs. The AUROC for discriminating pCR within the tumor compartment was 0.85 (95% CI 0.63–1.00) for CD11c^+^ (macrophage and DCs), 0.76 (95% CI 0.53–0.99) for CD11c^+^/MHCII^+^/CD163^–^/CD68^–^ (DCs), 0.80 (95% CI 0.54–1.00) for CD11c^+^/MHCII–/CD163–/CD68– (nonactivated/immature DCs), and 0.77 (95% CI 0.55–0.99) for CD11c^+^/CD163^+^ (M2 macrophage; Table 1). The CD11c/CD3 baseline ratio was 0.75 (95% CI 0.52–0.98) in the tumor compartment, 0.44 (95% CI 0.17–0.71) in the stromal compartment, and 0.58 (95% CI 0.29–0.87) in the total tumor (Table 1). Cell density trended higher in responders than in nonresponders across all immune subsets (Fig. 1). T-cell content, as measured by CD3^+^ cell density at baseline in the tumor and stromal compartments and total tumor trended higher in responders than in nonresponders. CD8^+^/Granzyme B^+^/Ki67^+^ also trended higher in responders compared with nonresponders, whereas overall CD8^+^ cell density was comparable between responders and nonresponders in both the tumor and stromal compartments and in the total tumor (Fig. 2). After one cycle of pembrolizumab, the baseline detrended change from baseline in size of the CD163^+^/MHCII^+^ population trended higher in nonresponders than in responders in the stromal compartment (AUROC, 0.2; 95% CI 0.0–0.42; Table 1; Fig. 3).

**Table 1:** AUROC (95% CI) for discriminating pCR based on immune subsets and spatial localization

AUROC (95% CI)	Tumor	Stromal	Total tumor
Cell population, baseline			
CD11c^+^ (macrophages and DCs)	0.85 (0.63 to 1.00)	–	–
CD11c^+^/MHCII^+^/CD163^–^/CD68^–^ (DCs)	0.76 (0.53 to 0.99)	–	–
CD11c^+^/MHCII^–^/CD163^–^/CD68^–^ (nonactivated/immature DCs)	0.80 (0.54 to 1.00)	–	0.76 (0.51 to 1.00)
CD11c^+^/CD163^+^ (M2 macrophages)	0.77 (0.55 to 0.99)	–	–
Cell population, baseline ratio			
CD11c/CD3 ratio	0.75 (0.52 to 0.98)	0.44 (0.17 to 0.71)	0.58 (0.29 to 0.87)
Detrended change from baseline and pCR after baseline^a^			
CD163^+^/MHCII^+^ (DC3)	0.58 (0.31 to 0.86)	0.20 (0 to 0.42)	0.26 (0.03 to 0.50)

*AUROC* area under the receiver operating characteristic curve, *CI* confidence interval, *DC* dendritic cell, *pCR* pathologic complete response

^a^Negative correlations between change from baseline and baseline values were observed; therefore, baseline de-trending was applied to change from baseline values

**Fig. 4:**
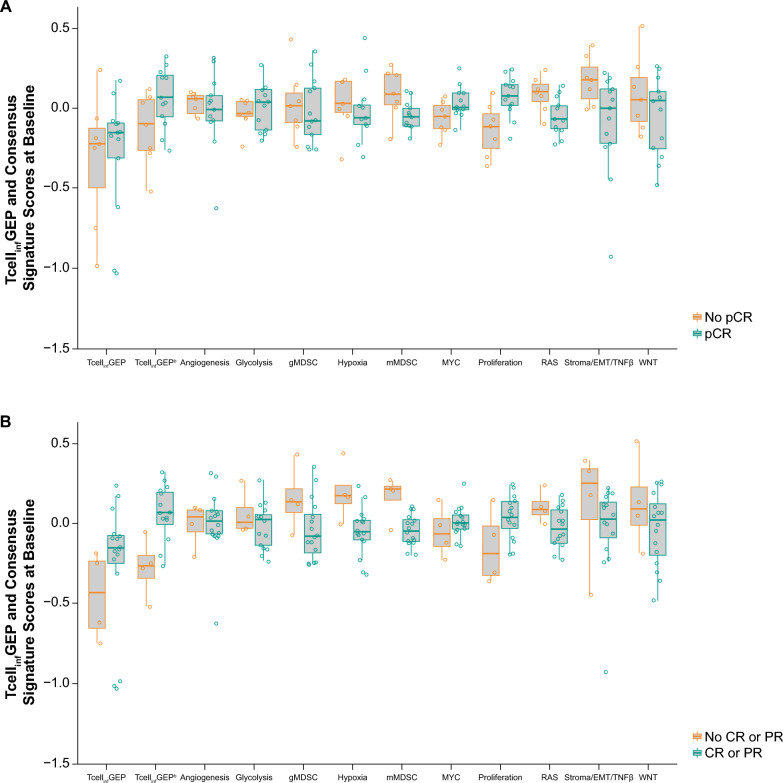
Tcell_inf_GEP and non-Tcell_inf_GEP^a^ consensus signature score at baseline by **A** pCR and **B** ORR. Abbreviations: CR: complete response; EMT: epithelial mesenchymal transition; gMDSC: granulocytic myeloid-derived suppressor cell; mMDSC: monocytic myeloid-derived suppressor cell; ORR: objective response rate; pCR: pathologic complete response; PR: partial response; Tcell_inf_GEP: T-cell–inflamed gene expression profile; TGFβ: transforming growth factor β. ^a^Detrended by Tcell_inf_GEP. ^b^Detrended by mMDSC

**Fig. 5:**
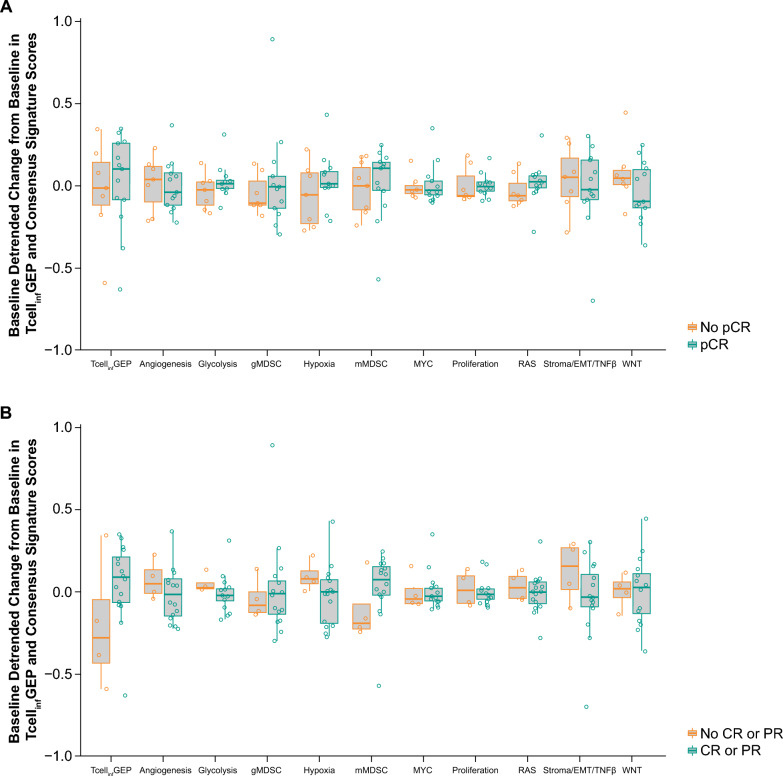
Tcell_inf_GEP and non-Tcell_inf_GEP consensus signature score detrended change from baseline by **A** pCR and **B** ORR. Abbreviations: CR: complete response; EMT: epithelial mesenchymal transition; gMDSC: granulocytic myeloid-derived suppressor cell; mMDSC: monocytic myeloid-derived suppressor cell; ORR: objective response rate; pCR pathologic complete response; PR: partial response; Tcell_inf_GEP: T-cell–inflamed gene expression profile; TGFβ: transforming growth factor β

The AUROC for discriminating pCR based on Tcell_inf_GEP was 0.55 (95% CI 0.25–0.85); when detrended by mMDSC, this was 0.75 (95% CI 0.51–0.98; Table 2). The AUROC for discriminating for ORR based on Tcell_inf_GEP was 0.75 (95% CI 0.54–0.96); when detrended by mMDSC, the AUROC was 0.92 (95% CI 0.78–1.00; Table 2). The AUROC for discriminating response was evaluated for each of the 10 non-Tcell_inf_GEP consensus signatures after detrending by Tcell_inf_GEP (Table 2). The AUROC (95% CI) for discriminating pCR based on proliferation, RAS, and stroma/EMT/TGFβ signatures and the AUROC (95% CI) for discriminating ORR based on gMDSC, hypoxia, mMDSC, and RAS signatures did not cross 0.5 (Table 2). Tcell_inf_GEP expression trended higher in responders than in nonresponders when evaluating pCR and ORR. Expression of gMDSC, hypoxia, mMDSC, RAS, stroma/EMT/TNFβ, and WNT trended lower in responders than in nonresponders; MYC and proliferation trended higher in responders than in nonresponders (Fig. 4). For patient-level response after one cycle of pembrolizumab, glycolysis and hypoxia signatures change from baseline after detrending by baseline were lower in responders as assessed by ORR (Fig. 5).

**Table 2:** AUROC (95% CI) for discriminating pCR and ORR based on Tcell_inf_GEP and non-Tcell_inf_GEP consensus signatures at baseline

AUROC (95% CI)	pCR	ORR
Tcell_inf_GEP	0.55 (0.25 to 0.85)	0.75 (0.54 to 0.96)
Tcell_inf_GEP detrended by mMDSC	0.75 (0.51 to 0.98)	0.92 (0.78 to 1.00)
Non-Tcell_inf_GEP consensus signature^a^		
Angiogenesis	0.40 (0.14 to 0.65)	0.45 (0.07 to 0.83)
gMDSC	0.41 (0.14 to 0.67)	0.20 (0.00 to 0.43)
Glycolysis	0.60 (0.34 to 0.87)	0.39 (0.10 to 0.69)
Hypoxia	0.37 (0.09 to 0.66)	0.14 (0.00 to 0.36)
mMDSC	0.22 (0.00 to 0.50)	0.12 (0.00 to 0.38)
MYC	0.75 (0.5 to 0.99)	0.66 (0.24 to 1.00)
Proliferation	0.86 (0.68 to 1.00)	0.73 (0.35 to 1.00)
RAS	0.20 (0.00 to 0.41)	0.22 (0.00 to 0.45)
Stroma/EMT/TGFβ	0.22 (0.01 to 0.43)	0.28 (0.00 to 0.72)
WNT	0.37 (0.11 to 0.64)	0.36 (0.02 to 0.70)

*AUROC* area under the receiver operating characteristic curve; *CI* confidence interval; *EMT* epithelial mesenchymal transition; *gMDSC* granulocytic myeloid-derived suppressor cell; *mMDSC* monocytic myeloid-derived suppressor cell; *ORR* objective response rate; *pCR* pathologic complete response; *Tcell*_*inf*_*GEP* T-cell–inflamed gene expression profile; *TGFβ* transforming growth factor β

^a^Detrended by Tcell_inf_GEP

## Discussion

In this exploratory analysis of patients with newly diagnosed early-stage TNBC who received neoadjuvant pembrolizumab plus chemotherapy in KEYNOTE-173, the strongest correlation of response was observed with immune subsets at baseline within the tumor compartment. Myeloid cell populations demonstrated multiple positive correlations with response. T-cell population correlations with response were relatively weak, although a stronger correlation was observed in one CD8^+^ subset. Tcell_inf_GEP expression trended higher in responders than in nonresponders (assessed by pCR), especially when detrended by mMDSC. The non-Tcell_inf_GEP signatures at baseline detrended by Tcell_inf_GEP were mostly consistent with the hypothesized direction in responders versus nonresponders assessed by both pCR and ORR, although slightly better AUROC was observed when evaluating ORR compared with pCR. Upon detrending from baseline, change with treatment in the CD163^+^/MHCII^+^ (DC3) population in the stromal compartment was the only immune subset for which the AUROC did not cross 0.5.

Use of PD-(L)1 inhibitors for the treatment of early-stage TNBC is supported by a growing body of evidence. The addition of pembrolizumab to neoadjuvant chemotherapy significantly improved pCR compared with standard chemotherapy in patients with early-stage high-risk TNBC across multiple clinical studies, including the randomized phase 3 KEYNOTE-522 study [12, 13, 18, 31]. In the phase 3 IMpassion031 study, neoadjuvant therapy with the PD-L1 inhibitor atezolizumab plus chemotherapy compared with chemotherapy also significantly improved pCR rates [15]. Neoadjuvant therapy with durvalumab plus chemotherapy and combination therapy with the PD-1 inhibitor nivolumab plus the cytotoxic T-lymphocyte–associated protein 4 inhibitor ipilimumab have also demonstrated improvement in survival and pCR, respectively, in smaller phase 2 studies [14, 16].

Similar to the present findings, higher levels of CD8^+^, Tcell_inf_GEP, and the glycolysis signature were associated with response to pembrolizumab plus chemotherapy in exploratory analyses of the KEYNOTE-086 study of patients with metastatic TNBC [19, 20]. In this study, immune cell density trended higher in the tumor compartment regardless of the immune subset for responders. The only immune subset that trended higher in the stroma and in the negative direction was CD163^+^/MHCII^+^. It is possible that suppressive or tolerizing myeloid populations may show an unexpected sensitivity to pembrolizumab plus chemotherapy in this setting, yielding a better clinical response. Notably, the higher pre-treatment myeloid populations within the tumor compartment associated with response was also part of the Tcell_inf_GEP signature and activated CD8^+^ T-cell subset analysis. The higher pre-treatment myeloid population may have also contributed to the trend of higher cell density in the tumor compartment regardless of response. A previous study found highest cell densities in the stroma compared with tumor compartments in TNBC, suggesting that pembrolizumab may exhibit selectivity in its action within the tumor compartment [32]. Presence of CD8 T cells distinctly located at the invasive tumor margin have also been associated with response to pembrolizumab therapy [9].

This analysis used the mIHC technique to characterize the TME and evaluate immune subsets that may be predictive of response to neoadjuvant pembrolizumab plus chemotherapy in TNBC. Currently, limited data exist on the evaluation of multiple biomarkers to define immune subsets by spatial localization in patients treated with a PD-(L)1 inhibitor plus chemotherapy. These analyses provide insight into the tumor heterogeneity of TNBC and the effects of one cycle of neoadjuvant pembrolizumab on immune subsets and gene expression. The key limitations of this study include the lack of a control group, exploratory nature of the analysis, and small sample size, which preclude definitive conclusions regarding the value of these biomarkers in predicting response to neoadjuvant pembrolizumab plus chemotherapy in early-stage high-risk TNBC. Additionally, the scalability of assessing immune subsets as biomarkers using mIHC remains a challenge; efforts to make these analyses scalable for implementation in the clinical setting are still needed.

Overall, the molecular characteristics most strongly associated with response included higher immune cell densities in the tumor compartment. The TME characteristics identified in this study are hypothesis generating and require further validation in larger studies.

## Supplementary Information

Additional file 1.

## Data Availability

Merck Sharp & Dohme LLC, a subsidiary of Merck & Co., Inc., Rahway, NJ, USA (MSD), is committed to providing qualified scientific researchers access to anonymized data and clinical study reports from the company’s clinical trials for the purpose of conducting legitimate scientific research. MSD is also obligated to protect the rights and privacy of trial participants and, as such, has a procedure in place for evaluating and fulfilling requests for sharing company clinical trial data with qualified external scientific researchers. The MSD data sharing website (available at http://engagezone.msd.com/ds_documentation.php) outlines the process and requirements for submitting a data request. Applications will be promptly assessed for completeness and policy compliance. Feasible requests will be reviewed by a committee of MSD subject matter experts to assess the scientific validity of the request and the qualifications of the requestors. In line with data privacy legislation, submitters of approved requests must enter into a standard data-sharing agreement with MSD before data access is granted. Data will be made available for request after product approval in the United States and the European Union or after product development is discontinued. There are circumstances that may prevent MSD from sharing requested data, including country- or region-specific regulations. If the request is declined, it will be communicated to the investigator. Access to genetic or exploratory biomarker data requires a detailed, hypothesis-driven statistical analysis plan that is collaboratively developed by the requestor and MSD subject matter experts; after approval of the statistical analysis plan and execution of a data-sharing agreement, MSD will either perform the proposed analyses and share the results with the requestor or will construct biomarker covariates and add them to a file with clinical data that is uploaded to an analysis portal so that the requestor can perform the proposed analyses.

## Abbreviations

AUC: Area under the curve
AUROC: Area under the receiver operating characteristic curve
CI: Confidence interval
DC: Dendritic cell
EMT: Epithelial mesenchymal transition
gMDSC: Granulocytic myeloid-derived suppressor cell
HER2: Human epidermal growth factor receptor-2
HR: Hazard ratio
mIHC: Multiplex immunohistochemistry
mMDSC: Monocytic myeloid-derived suppressor cell
ORR: Objective response rate
pCR: Pathologic complete response
PD-(L)1: Programmed cell death protein 1 or programmed death ligand 1
Q3W: Every 3 weeks
Tcell_inf_GEP: T-cell–inflamed gene expression profile
TGFβ: Transforming growth factor β
TME: Tumor microenvironment
TNBC: Triple-negative breast cancer
