# Supplementary material for: Molecular determinants of response to neoadjuvant pembrolizumab plus chemotherapy in patients with high-risk, early-stage, triple-negative breast cancer: exploratory analysis of the open-label, multicohort phase 1b KEYNOTE-173 study
Source: Breast Cancer Res. 2025 Mar 11;27:35. doi: 10.1186/s13058-024-01946-y (PMC11895130; doi:10.1186/s13058-024-01946-y)
Supplement: Supplementary file 1 — Additional file 1. [file 13058_2024_1946_MOESM1_ESM.docx]

**SUPPLEMENTAL MATERIAL**

**Supplementary Table S1.** Primary antibodies used for mIHC

| **Antibody** | **Host/clone** | **Supplier** | **Paired opal fluorophore** |
| --- | --- | --- | --- |
| CD103 | Rabbit/EPR4166 | Abcam, Boston, MA, USA | Opal480 |
| CD39 | Mouse/2B10 | Lifespan Biosciences, Seattle, WA, USA | Opal520 |
| Granzyme B | Mouse/GZB01 | Thermo Fisher Scientific, Fremont, CA, USA | Opal570 |
| CD8 | Mouse/C8/144B | Agilent, Bayonne, NJ, USA | Opal620 |
| Cytokeratin | Mouse/AE1/AE3 | Agilent, Bayonne, NJ, USA | Opal690 |
| Ki67 | Mouse/MIB-1 | Agilent, Bayonne, NJ, USA | Opal780 |
| MHCII (HLA-DR) | Mouse/LN3 | Thermo Fisher Scientific, Fremont, CA, USA | Opal690 |
| Foxp3 | Mouse/236A/E7 | Abcam, Boston, MA, USA | Opal480 |
| CD163 | Mouse/10D6 | Thermo Fisher Scientific, Fremont, CA, USA | Opal520 |
| Fibroblast activation protein | Rabbit/EPR20021 | Abcam, Boston, MA, USA | Opal570 |
| CD68 | Mouse/KP1 | Agilent, Bayonne, NJ, USA | Opal620 |
| CD11c | Mouse/5D11 | Leica Biosystems, Deer Park, IL, USA | Opal780 |

Abbreviations: mIHC: multiplex immunohistochemistry.
